# A fully featured COMBINE archive of a simulation study on syncytial mitotic cycles in *Drosophila *embryos

**DOI:** 10.12688/f1000research.9379.1

**Published:** 2016-09-29

**Authors:** Martin Scharm, Dagmar Waltemath

**Affiliations:** 1 Department of Systems Biology and Bioinformatics, Institute of Computer Science, University of Rostock, Rostock, Germany

**Keywords:** COMBINE, data, containers

## Abstract

COMBINE archives are standardised containers for data files related to a simulation study in computational biology. This manuscript describes a fully featured archive of a previously published simulation study, including (i) the original publication, (ii) the model, (iii) the analyses, and (iv) metadata describing the files and their origin. With the archived data at hand, it is possible to reproduce the results of the original work. The archive can be used for both, educational and research purposes. Anyone may reuse, extend and update the archive to make it a valuable resource for the scientific community.

## Introduction

In systems biology and systems medicine, the steadily increasing size and complexity of simulation studies pose additional challenges to sharing reproducible results ^1^. Repeated mentions of problems with replication and reproducibility ^2– 4^ led to new standards, tools, and methods for the transfer of reproducible simulation studies ^5– 9^. Several projects and initiatives already deal with reproducibility issues, such as COMBINE ( co.mbine.org), FAIRDOM ( fair-dom.org), and the Reproducibility Initiative ( reproducibilityinitiative.org).

The *Computational Modeling in Biology Network* (COMBINE) coordinates the development of standard formats for various aspects of a simulation study: The Systems Biology Markup Language (SBML) ^10^ and CellML ^11^ encode the mathematical models; the Systems Biology Graphical Notation (SBGN) ^12^ encodes the visual representation of models; the Simulation Experiment Description Markup Language (SED-ML) ^13^ encodes the simulation recipes; and the Systems Biology Result Markup Language (SBRML) ^14^ encodes numerical data and simulation results.

Today’s studies consist of multiple, heterogeneous, and sometimes distributed data files, leading to the challenge of exchanging complete and thus reproducible results. To close this gap, the COMBINE community developed the COMBINE archive ^8^. A COMBINE archive is a single file that aggregates all data files and information necessary to reproduce a simulation study in computational biology. The skeleton of a COMBINE archive consists of a manifest and a metadata file, specified by the Open Modeling EXchange format (OMEX).

Here we describe a fully featured COMBINE archive, which encodes an investigation of the syncytial mitotic cycles in *Drosophila* embryos ^15^. The study published by Calzone *et al.* proposes a dynamical model for the molecular events underlying rapid, synchronous, syncytial nuclear division cycles in *Drosophila* embryos. This particular study was chosen for several reasons. Firstly, the paper, the documentation, and the related data are openly accessible. Secondly, the model is available in two standard formats: The CellML encoding is available from the Physiome Model Repository ^16^ at models.cellml.org/exposure/1a3f36d015121d5596565fe7d9afb332 and the SBML encoding is available from BioModels ^17^ at www.ebi.ac.uk/biomodels-main/BIOMD0000000144. Thirdly, both model files are already curated, which increases the level of trust. Fourthly, the model describes a common biological system (cell cycle). Thus, the basic mechanisms of the encoded biology should be familiar to many researchers, reducing the effort of understanding the example.

This archive contains files that are openly available for download, as well as previously unpublished files that were generated using COMBINE-compliant software tools (see Section Materials and methods). When executed, it reproduces the original findings by Calzone *et al.*

## Materials and methods

The fully featured COMBINE archive was created in three subsequent steps. Firstly, all available materials relating to the study were automatically retrieved from an online resource (initial archive). Secondly, the data files were organised into subdirectories, following the different aspects of a simulation study (documentation, model, experiment, result). Thirdly, missing files were manually retrieved from web resources or created using COMBINE-compliant software tools. The three steps are described in the following.

### Retrieving an initial COMBINE archive

The initial version of the COMBINE archive was generated using the web-based software tool M2CAT ^18^ Version 0.1 ( m2cat.sems.uni-rostock.de). Among the suggested archives for the work by Calzone *et al.*, we chose the simulation study containing a CellML model and a visualisation of the model in three different formats (PNG, SVG, AI). M2CAT automatically generated the initial COMBINE archive from these files. It also added metadata to the archive, such as annotations to creators, contributors, and modification times. M2CAT retrieved this metadata from the corresponding GIT project in the Physiome Model Repository ( git log).

### Organising the COMBINE archive

For convenience, the files inside the COMBINE archive were structured in subfolders. The initial archive was therefor imported into the CombineArchiveWeb application (WebCAT, ^9^) Version 0.4.13 ( webcat.sems.uni-rostock.de). WebCAT is a web interface to display and modify the files contained in an archive, together with metadata and file structures. The files inside the archive were organised in four directories, which reflect the different aspects of a simulation study:

• documentation/: files that describe and document the model and/or experiment ( *empty*)
• model/: files that encode and visualise the biological system ( *4 files*)
• experiment/: files that encode the *in silico* setup of the experiment ( *empty*)
• result/: files that result from running the experiment ( *empty*)

All files in the initial archive were stored in the model/ directory. However, these files alone are not sufficient to reproduce the study.

### Extending the COMBINE archive

To make the encoded study reproducible, the COMBINE archive needs to be extended with additional files.

**The article** is typically the central object of a research study. For this study, the original publication by Calzone *et al.*, together with available supplementary information, was retrieved from the homepage of the journal *Molecular Systems Biology* ( msb.embopress.org/content/3/1/131). Using WebCAT, the files were uploaded to the documentation/directory of the archive. The automatically added metadata was adjusted to attribute the authors of the publication and to state when and where the files were downloaded. In the background, WebCAT encoded the metadata in RDF/XML and added it to the archive.

**The model** is not only available in CellML format, but also in SBML format. The SBML file was retrieved from BioModels ( www.ebi.ac.uk/biomodels-main/download?mid=BIOMD0000000144, SBML Level 2 Version 1) and uploaded to the model/directory. Again, the metadata was corrected to attribute the original authors, curators, and contributors, as stated on the BioModels website ( www.ebi.ac.uk/biomodels-main/BIOMD0000000144) and in the model document.

**Figure 1.**
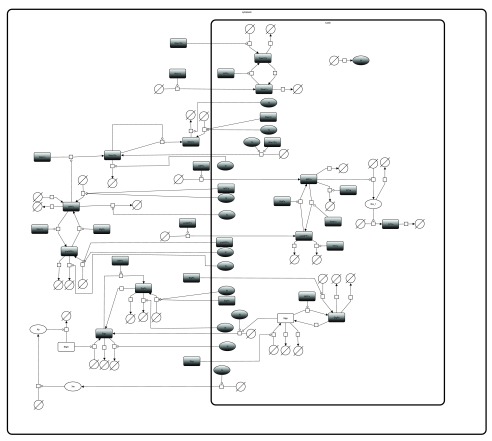
Visualisation of the model. This figure shows the SBGN-PD compliant reaction network, as encoded in the SBML model obtained from BioModels. The figure was generated and modified using SBGN-ED.

**The simulation description** is essential to run the experiment. It defines the simulation environment and the output of the *in silico* execution. As no simulation description was found in any of the open repositories, an initial version was created using the SED-ML Web Tools (SWT) Version 2.1 ( bqfbergmann.dyndns.org/SED-ML_Web_Tools). SWT takes the model files and creates a default simulation description with standard settings. For this study, a default SED-ML file encodes instructions to generate 66 plots and a data table. Each plot describes the change of concentration in one species of the model. The data table contains all numerical values. Based on the default script, a second SED-ML file ( Calzone2007-simulation-figure-1B.xml) was generated to reassemble Figure 1B of the original publication. Using WebCAT, both SED-ML scripts were added to the experiment/directory of the archive. The metadata for the new files was added.

**The simulation results** reflect the behaviour of a model under certain conditions. The script defined in Calzone2007-simulation-figure-1B.xml was loaded into SWT and into the stand-alone software program COPASI Version 4.15 Build 95 ^19^. The plots generated by both tools show that the developed *in silico* experiment reproduces the results from the paper. Using WebCAT, the figures produced by SWT and COPASI were uploaded and added to the result/ directory of the archive. Metadata, such as the versions of the software tools, was added accordingly.

**The visualisation of a model** helps to understand the encoded biological system. For this study, an SBGN-compliant visualisation of the model was created using SBGN-ED Version 1.5.1 ^20^ together with VANTED Version 2.1.0 ^21^. SBGN-ED generated an automatic layout of the uploaded SBML model, which was then improved manually. The resulting Figure 1 was exported in different formats (GraphML ^22^, GML ( www.fim.uni-passau.de/index.php?id=17297&L=1), PNG image, PDF, and SBGN-ML ^23^). Using WebCAT the files were uploaded to the model/sbgn directory and metadata was provided.

## Data description

The archive consists of 21 files ( Table 1). Among these files are the manifest.xml and the metadata.rdf, which form the skeleton of the archive. The manifest lists the files included in the archive. The metadata file contains additional information about the files in the archive, such as creators and descriptions. A third file, README.md, contains a description for visitors of the GitHub repository, where the archive is being developed ( github.com/SemsProject/CombineArchiveShowCase). The remaining 18 files are organised in four directories, cmp. Section Organising the COMBINE archive. The original publication (PDF) is stored in the documentation/ directory. The encodings of the model (CellML, SBML, graph formats) are stored in the model/ directory. The simulation descriptions (SED-ML) are stored in the experiment/ directory. The simulation results (SVG, PNG) are stored in the result/ directory.

**Table 1.** Content of the fully featured COMBINE archive. The table lists all files included in the presented COMBINE archive together with formats and descriptions. The indentation indicates the directory structure used to organise the files in the archive.

File	Format	Description
manifest.xml	Omex	Skeleton, automatically generated by WebCAT
metadata.rdf	Omex	Skeleton, automatically generated by WebCAT
README.md	Markdown	Human readable information for users stumbling upon the archive
model/		
BIOMD0000000144.xml	SBML L2V1	origin: www.ebi.ac.uk/biomodels-main/download?mid=BIOMD0000000144
calzone_2007.svg	SVG	origin: models.cellml.org/workspace/calzone_thieffry_tyson_novak_2007
calzone_2007.ai	Illustrator	origin: models.cellml.org/workspace/calzone_thieffry_tyson_novak_2007
calzone_2007.png	PNG	origin: models.cellml.org/workspace/calzone_thieffry_tyson_novak_2007
calzone_thieffry_tyson_novak_2007.cellml	CellML 1.0	origin: models.cellml.org/workspace/calzone_thieffry_tyson_novak_2007
sbgn/Calzone2007.gml	GML	SBGN compliant figure generated using SBGN-ED
sbgn/Calzone2007.graphml	GraphML	SBGN compliant figure generated using SBGN-ED
sbgn/Calzone2007.pdf	PDF	SBGN compliant figure generated using SBGN-ED
sbgn/Calzone2007.png	PNG	SBGN compliant figure generated using SBGN-ED
sbgn/Calzone2007.sbgn	SBGN-ML	SBGN-ML encoded figure generated using SBGN-ED
experiment/		
Calzone2007-default-simulation.xml	SED-ML L1V1	Simulation description generated using SED-ML Web Tools
Calzone2007-simulation-figure-1B.xml	SED-ML L1V1	Simulation description generated using SED-ML Web Tools based on Calzone2007-default-simulation.xml
documentation/		
Calzone2007.pdf	PDF	Scientific publication *“Dynamical modeling of syncytial mitotic cycles in* *Drosophila embryos”* obtained from msb.embopress.org/content/3/1/131
Calzone2007-supplementary-material.pdf	PDF	Supplementary information for the publication obtained from msb.embopress.org/content/3/1/131
result/		
Fig1B-bottom-COPASI.svg	SVG	Image generated by executing Calzone2007-simulation-figure-1B.xml on BIOMD0000000144.xml in COPASI
Fig1B-top-COPASI.svg	SVG	Image generated by executing Calzone2007-simulation-figure-1B.xml on BIOMD0000000144.xml in COPASI
Fig1B-bottom-webtools.png	PNG	Image generated by executing Calzone2007-simulation-figure-1B.xml on BIOMD0000000144.xml in SED-ML Web Tools
Fig1B-top-webtools.png	PNG	Image generated by executing Calzone2007-simulation-figure-1B.xml on BIOMD0000000144.xml in SED-ML Web Tools

The latest version of the compiled COMBINE archive can be accessed through our web server at scripts.sems.uni-rostock.de/getshowcase.php.

## Data validation

**Figure 2.**
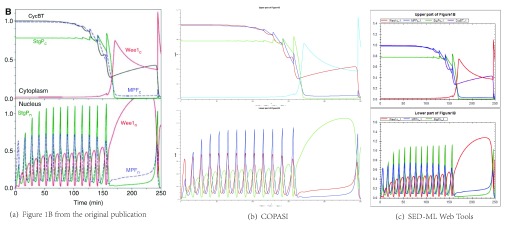
Comparison of simulation results. The figure shows the simulation results included in the original publication ( **2(a)**). Furthermore, the results generated by COPASI ( **2(b)**) and the SWT ( **2(c)**), using the SED-ML script Calzone2007-simulation-figure-1B.xml, are shown.

The COMBINE archive described in this data note reproduces the results of the study published by Calzone *et al.* To validate the reproducibility, we executed the archive in different simulation tools. For example, the encoded simulation study can be executed in COPASI, cmp. Figure 2(b). The archive can also be loaded to the SWT by opening a specific URL ( bqfbergmann.dyndns.org/SED-ML_Web_Tools/Home/SimulateUrl?url=http://scripts.sems.uni-rostock.de/getshowcase.php). The simulation results will immediately be shown in the web browser, cmp. Figure 2(c). Moreover, users reported a successful reproduction of the simulation results using Tellurium ^24^ ( github.com/SemsProject/CombineArchiveShowCase/pull/2).

## Conclusions

The presented COMBINE archive provides a reproducible simulation study for a previously published model on syncytial mitotic cycles in *Drosophila* embryos ^15^. The archive contains several files that were collected from online resources, e. g. the CellML model from the Physiome Model Repository or the scientific publication from the publisher’s website. It also provides new files that did not exist previously, e. g. a SED-ML file to encode the simulation setup for Figure 1B of the original publication.

This fully featured archive allows scientists to reproduce the results obtained by Calzone *et al.* in software tools that can read COMBINE archives. For example, the archive was successfully executed in the SED-ML Web Tools and Tellurium. Figure 2 shows that the developed study is able to reproduce the original results.

This data note describes the fully featured COMBINE Archive as published on Figshare ^25^. However, we expect the archive to evolve further. The latest version of the archive is available from GitHub at github.com/SemsProject/CombineArchiveShowCase. It can also be downloaded from our website at scripts.sems.uni-rostock.de/getshowcase.php. Extensions, refinements, and comments are very welcome. Please fork the project on GitHub and contribute pull requests.

## Data availability

The data referenced by this article are under copyright with the following copyright statement: Copyright: © 2016 Scharm M and Waltemath D

Data associated with the article are available under the terms of the Creative Commons Zero "No rights reserved" data waiver (CC0 1.0 Public domain dedication). 

The latest version of the COMBINE archive: github.com/SemsProject/CombineArchiveShowCase/ (latest commit at the time of submission: a469197)

The fully featured COMBINE archive as at the time of publication: *Figshare*: COMBINE Archive Show Case, 10.6084/m9.figshare.3427271.v1 ^24^
