# Supplementary material for: A fully featured COMBINE archive of a simulation study on syncytial mitotic cycles in Drosophila embryos
Source: F1000Res. 2016 Sep 29;5:2421. [Version 1] doi: 10.12688/f1000research.9379.1 (PMC5082596; doi:10.12688/f1000research.9379.1)
Supplement: Peer review history

# Peer review: Referee response for version 1

**DOI:** 10.5256/f1000research.10101.r16693

**Published:** 2016-10-11

**Authors:** Alan Garny

**Affiliations:** 1 University of Auckland, Auckland, New Zealand

A major issue in computational biology is that of reproducibility. Here, the authors provide us with a concrete example of a reproducible simulation experiment using the Calzone *et al.* model (2007) of syncytial mitotic cycles in *Drosophila* embryos. To achieve their aim, they use established mark-up languages such as CellML, SBML and SED-ML, and package everything in a COMBINE archive. This COMBINE archive can then be used by anyone to reproduce the simulation experiment, as well as have access to the original paper, SBGN diagram, etc. This data note therefore shows us one possible, and viable, way to address the issue of reproducibility in computational biology and should, as such, be considered for indexing.

**Major comments:**

- How do you envisage tackling the issue of provenance? Say that someone modifies your archive and makes it available to the community, how are we then supposed to know which one to use and what the differences are between the two versions? Considering that your COMBINE archive is on GitHub, I guess someone could always fork it, but maybe a better approach would be to take advantage of existing repositories in the community, such as BioModels.net and the Physiome Model Repository (PMR)? I am not sure about BioModels.net, but PMR inherently addresses the issue of provenance.
- In their supplementary material, Calzone *et al.* provide some files that can be used to reproduce their different figures. So, in effect, they allow for their results to be reproduced (using XPPAUT). It would therefore make sense to have a comparison of their ‘approach’ to reproducibility compared with yours.
- The two SED-ML files in your COMBINE archive refer to the SBML version of the Calzone model. Now, because you are also providing a CellML version of that model, it would be nice to also have a CellML-based version of your two SED-ML files.

 

**Minor comments:**

- **Abstract:**
  - You might want to replace “previously published simulation study” with “model of syncytial mitotic cycles in *Drosophila* embryos”.
- **Keywords:**
  - Apparently, authors should supply up to eight keywords.
- **Introduction:**
  - **1 ^st^ paragraph:** COmputational Modeling in BIology NEtwork (i.e. “O” instead of “o” and “E” instead of “e”).
  - **2 ^nd^ paragraph:** “the Systems Biology…” and not “The Systems Biology…”?
  - **2 ^nd^ paragraph:** wouldn’t it be better to reference NuML rather than SBRML? (On http://co.mbine.org/Standards, SBRML is only mentioned as part of the NuML description.)
  - **4 ^th^ paragraph:** “Here, we describe…” (i.e. missing comma).
- **Materials and methods:**
  - **Retrieving an initial COMBINE archive:**
    - It would be nice to know exactly what kind of search was done using M2CAT (probably one using the term “calzone”?) and, then, which COMBINE archive was used as an initial COMBINE archive.
  - **Organising the COMBINE archive:**
    - “The initial archive was therefor[e] imported…” (therefore).
    - It would be nice to know what those 4 initial files were (those in the *model* folder).
  - **Extending the COMBINE archive:**
    - **The article:** “…to state when and [from] where…” (from).
    - **The simulation description:**
      - What is the point of having that default simulation experiment? To use it as a starting point for reproducing Figure 1B of the Calzone paper is fine, but I don’t see the point of including that default simulation experiment in the COMBINE archive.
      - It would be nice to know what simulation parameters and solver (incl. its parameters) were used to reproduce Figure 1B of the Calzone paper. (I imagine they are the same as the ones used by the authors with XPPAUT?)
      - You might want to use sysbioapps.dyndns.org/SED-ML_Web_Tools as the URL for SED-ML Web Tools (rather than bqfbergmann.dyndns.org/SED-ML_Web_Tools)?
      - It would be nice to have all the other figures of the Calzone paper also encoded in SED-ML, just so that your COMBINE archive is not only fully featured, but also complete.
    - The visualisation of a model:
      - Are you sure about the version of VANTED you used? Version 2.1.0 is somewhat old compared with the latest version available (version 2.6.3). Actually, looking at the contents of your COMBINE archive, I can see that Calzone2007.gml was generated using VANTED 2.6.2. (FWIW, SBGN-ED 1.6 has just been released.)
      - The link for GML is broken ( http://www.fim.uni-passau.de/index.php?id=17297&L=1).
- **Data description:**
  - “…in four directories, cmp. Section…”?
  - Rather than referring to scripts.sems.uni-rostock.de/getshowcase.php (in this section and elsewhere in the manuscript), you might want to refer to your GitHub repository ( github.com/SemsProject/CombineArchiveShowCase) and make use of GitHub’s release feature? It might be safer in the long term.
- **Data validation:**
  - “…can be executed in COPASI, cmp. Figure 2(b).”?
  - To click on the bqfbergmann.dyndns.org/SED-ML_Web_Tools/Home/SimulateUrl?url=http://scripts.sems.uni-rostock.de/getshowcase.php link takes me to a page that reads “No model uploaded. You need to upload a model first prior to attempting to simulate it!”
  - “… shown in the web browser, cmp. Figure 2(c).”?
  - You might want to provide a URL for COPASI and Tellurium?
- **Data availability:**
  - You might want to reference your very latest commit (i.e. 6c34cc4) rather than commit a469197?
- **Figure 1:**
  - I am not sure how useful this figure is. To me, it doesn’t bring anything to your data note. Not only that, but at its original size, one cannot read anything (I personally had to view it at 400% to be able to start reading the different labels).
- **Figure 2:**
  - You might want to remove the “B” in panel (a). It’s confusing.
  - You might want to mention that the range of the X and Y axes, as well as the colour of the different plots, cannot currently be specified in SED-ML, hence panels (b) and (c) don’t perfectly match panel (a)?

I have read this submission. I believe that I have an appropriate level of expertise to confirm that it is of an acceptable scientific standard, however I have significant reservations, as outlined above.

# Peer review: Referee response for version 1

**DOI:** 10.5256/f1000research.10101.r16691

**Published:** 2016-10-10

**Authors:** Laurence Calzone, Mihaly Koltai

**Affiliations:** 1 Institut Curie, Paris, France; 2 Computational Systems Biology of Cancer group, Institut Curie, Paris, France

The article describes a COMBINE archive of a previously published ODE model (Calzone et al) of the oscillatory dynamics of cell cycle regulatory proteins in Drosophila embryos undergoing rapid syncytial nuclear division cycles.

 The files are stored on GitHub in 4 subfolders, containing the original article, model files, the figures generated from the simulations, as well as metafiles. The XML and CellML files that were originally published along with the article were modified, by adding metadata on the authors.

 Issues:

- There is a clear advantage to such an archive for publication of such models, reproducibility etc., but the authors do not insist on it. This type of archive could be added to publications, and provided by the authors, with a full documentation. This more general aim of the article should be more forcefully and clearly stated.
- We tried to open the model file available on github (XML) using COPASI (4.16) but some errors appeared. We downloaded the initial model from Biomodels and it worked. Same issues with CellML files.
- The link pointing to the website "bqfbergmann.dyndns.org/SED-ML_Web_Tools/" is broken, but if copy-pasted the figures are correctly displayed.

We have read this submission. We believe that we have an appropriate level of expertise to confirm that it is of an acceptable scientific standard.

# Peer review: Referee response for version 1

**DOI:** 10.5256/f1000research.10101.r16695

**Published:** 2016-10-03

**Authors:** Lars Juhl Jensen

**Affiliations:** 1 NNF Center for Protein Research, University of Copenhagen, Copenhagen, Denmark

**General comments:**

 The data note describes how COMBINE is used to make available a mathematical model for simulation of syncytial mitotic cycles in Drosophila. Although well written, the aim of the article is somewhat unclear to me.

 The way the introduction is written and the archive is named suggest that the purpose of making the archive is to serve as a showcase for how to use COMBINE archives. However, the way the rest of the article is written focuses very much on how this specific archive was made and how it is organized. If the goal is to showcase COMBINE archives, I feel the manuscript lacks a description of not only how the specific archive was made but also why this would be a good way to make such archives in general. If, on the other hand, the aim is to make the specific model available, it lacks some more background on the specific model and possibly suggestions or examples of how it can be used.

**Major comments:**

 I am confused about what is to be considered the primary repository / access point. The article refers to both a GitHub repository, an omex file on figshare, and a link to a php script on their own server where one can retrieve the latest version of the omex file. This leads to a number of questions:

1. Am I right that GitHub is the primary place where development is done and improvements will made?
2. Is the omex file provided by the php script always up-to-date with the GitHub repository, or may the newest version on GitHub be even newer than the latest version made available as an omex file?
3. Will the omex file on figshare be updated, or will there only be a v1 there?

Considering that figshare has both versioning and API for submission, I think the best solution would be to abandon the php script. This would eliminate redundancy, as the omex file would only be on figshare. Since figshare has both versioned and version-less DOIs for datafiles, this would allow the authors to provide a stable DOI that always points to the latest version of the omex file and at the same time allow users to always cite the specific versioned DOI of the omex file they used in their work. Given that figshare has a submission API, it should be possible to make sure that the omex file is automatically updated based on the GitHub repository whenever needed.

**Technical issues:**

 Accessing the URL "bqfbergmann.dyndns.org/SED-ML_Web_Tools/Home/SimulateUrl?url=http://scripts.sems.uni-rostock.de/getshowcase.php" by copy and paste works correctly. However, clicking the link within the article instead opens "http://bqfbergmann.dyndns.org/SED-ML_Web_Tools/Home/Simulate" and leads to an error page instead of the promised results.

 The link "www.ebi.ac.uk/biomodels-main/download?mid=BIOMD0000000144" is similarly broken. When clicked, it instead tries to open "https://f1000research.com/articles/5-2421/www.ebi.ac.uk/biomodels-main/download?mid=BIOMD0000000144", which leads to an error.

 The URL "www.fim.uni-passau.de/index.php?id=17297&L=1" leads me to an error page irrespective of whether I copy it or click it.

**Minor corrections:**

 The authors several times use the abbreviation "cmp." to refer to other parts/figures in the article. I have never encountered this abbreviation before and was unable to find any other articles that use it. Given the context, I suspect "cf." may be what is meant.

I have read this submission. I believe that I have an appropriate level of expertise to confirm that it is of an acceptable scientific standard, however I have significant reservations, as outlined above.
